# Experiences on Integrated Personal Health Systems—evidence from eight European countries

**Published:** 2012-06-15

**Authors:** Maria Lluch

**Affiliations:** IPTS-JRC, European Commission, Spain

**Keywords:** telehealth, telecare, governance, impact, diffusion of innovation, European countries, Integrated Personal Health Systems

## Abstract

**Introduction:**

The current and projected rise in the ageing population and the resulting increase in chronic diseases at a time of reduced public budget expenditure are squeezing European healthcare systems. Multiple initiatives to address identified challenges emerged in Europe and serve as a test-bed for a set of policies promoting integrated care at regional and national level. England for instance with the forthcoming results on the WSD and the launch of the DALLAS programme aims to promote the use of ICT in chronic and fragile patients.

**Aims:**

The aim of this article is to explore the role of IPHS (Integrated Personal Health Systems) in delivering integrated care in 8 selected European Member States using a three-axis framework: Innovation, Governance and Impact.

**Methods:**

Reflecting the diversity of healthcare delivery models, eight European Member States were selected for in-depth study, namely: Denmark, Estonia, France, Germany, Italy, the Netherlands, Spain and the UK. In each of the countries, a set of experiences involving IPHS applications were identified and analysed following a regional approach.

**Results:**

Experiences in most of the countries targeted reflect a trend moving away from pilot projects towards IPHS large scale-deployment at regional level aiming at potentially widespread diffusion at national level. Many identified drivers and barriers as well as push and pull factors are common across experiences and settings. Others are specific to each setting due to contextual factors and the nature of each health and social care system. The experiences were analysed using the three-axis framework: Innovation, Governance and Impact. Findings from an innovation perspective, for instance, identified interoperability as a barrier to be overcome. A trend towards convergence between telecare and telehealth applications was also identified. From a governance perspective, a variety of issues to be addressed was identified. Defining appropriate incentives aligned across tiers of care was identified as a governance issue to be addressed. Some experiences in Italy or Andalucía (Spain) provide interesting approaches in this regard. Further, the lack of an enabling legal framework was often translated into liability concerns for healthcare professionals. In turn, this translated into higher resistance to adopt these technologies. From an impact perspective, although evidence is often limited, this does not always prevent stakeholders from wider IPHS deployment. In addition, in some cases (i.e. Catalonia), the involvement of the HTA agency has assisted in the evaluation and dissemination of results. Thus, these organisations can play a relevant role in promoting IPHS adoption by introducing rigour into the evaluation methods and by consolidating evidence on the topic.

**Conclusions:**

Although IPHS deployment remains limited, evidence shows that the IPHS sector is gaining momentum in Europe. The research approach using regions as the unit of analysis proved to be satisfactory. Even in countries where healthcare is highly centralised (such as France) or where strong market features in healthcare exist (such as the Netherlands), IPHS deployment seemed to be spreading following a regional model. For those interested in promoting IPHS applications, lessons learnt and policy interventions are suggested drawing on the findings.

